# Scoping out urban areas of tourist interest though geolocated social media data: Bucharest as a case study

**DOI:** 10.1007/s40558-022-00235-8

**Published:** 2022-10-17

**Authors:** Almudena Nolasco-Cirugeda, Clara García-Mayor, Cristina Lupu, Alvaro Bernabeu-Bautista

**Affiliations:** 1 Urban Design and Regional Planning Unit, Building Sciences and Urbanism Department, University of Alicante, Carretera San Vicente del Raspeig s/n, San Vicente del Raspeig, 03690 Alicante, Spain; 2 Department of Geography, Alexandru Ioan Cuza University, UAIC Corp B, Bulevardul Carol I nr. 20A, 700506 Iași, Romania

**Keywords:** Urban tourism, Areas of interest, Location-based social networks, TripAdvisor, Foursquare, Bucharest, Tourist cities

## Abstract

Social media data has frequently sourced research on topics such as traveller planning or the factors that influence travel decisions. The literature on the location of tourist activities, however, is scarce. The studies in this line that do exist focus mainly on identifying points of interest and rarely on the urban areas that attract tourists. Specifically, as acknowledged in the literature, tourist attractions produce major imbalances with respect to adjacent urban areas. The present study aims to fill this research gap by addressing a twofold objective. The first was to design a methodology allowing to identify the preferred tourist areas based on concentrations of places and activities. The tourist area was delimited using Instasights heatmaps information and the areas of interest were identified by linking data from the location-based social network Foursquare to TripAdvisor’s database. The second objective was to delimit areas of interest based on users’ existing urban dynamics. The method provides a thorough understanding of functional diversity and the location of a city’s different functions. In this way, it contributes to a better understanding of the spatial distribution imbalances of tourist activities. Tourist areas of interest were revealed via the identification of users’ preferences and experiences. A novel methodology was thus created that can be used in the design of future tourism strategies or, indeed, in urban planning. The city of Bucharest, Romania, was taken as a case study to develop this exploratory research.

**Supplementary Information:**

The online version contains supplementary material available at 10.1007/s40558-022-00235-8.

## Introduction

Urban tourism has proved to be a stimulating economic activity in European cities as well as a driving force of urban economies, over the last decades. Different indicators illustrate this phenomenon, such as an increase of over 14% in the number of overnight stays between 2012 and 2016—European Cities Marketing Benchmarking Report (Önder et al. 2018)—and the over 10% growth in the number of beds available in urban settings (Boivin and Tanguay 2019). The COVID-19 travel restrictions affected this tendency in 2020, but the time has come for cities to set their post-pandemic tourism recovery goals, despite new uncertainties (UNWTO 2022).

Cities are dynamic environments where human activity density is a core subject to continual change in socioeconomic processes (Krehl et al. 2016). In this way, the distribution of human activities is a major driver in the transformation of urban structures, playing a key role in city-planning decision-making. In this context, social media data provide a unique and advanced approach to portray people's spatiotemporal preferences and mobility patterns (Cheng et al. 2011). Specifically, location-based social network data—hereafter, LBSN data—have become an ever more useful complementary source to approach socio-spatial user behaviours and to analyse different urban dynamics (Martí et al. 2019b).

It is worth noting that at the beginning of 2020, there were over 470.5 million active social media users in Europe. Approximately 55% of the European population shares information via social media networks, as analysed in the Digital 2020: Global Digital Overview (Kemp 2020). In fact, that percentage has risen by 23.3% since 2020 (We Are Social 2022). The increasing number of social media users providing geolocated virtual footprints is particularly suitable for urban research (Arribas-Bel 2014), facilitating a multiscale approach to spatial distribution patterns (van Meeteren and Poorthuis 2018).

Social media has become a key tool for the travel industry and has been used in an array of studies, from the identification of tourist segments through user-generated content—UGC—(Hernández et al. 2018), to the influence of online-tourist-community behaviours (Miguéns et al. 2008). Moreover, UGC is one of the three primary sources of tourism-related big data studies, the other two sources being the information provided by devices and operations (Li et al. 2018; Xu et al. 2019).

Indeed, data retrieved from LBSNs and other social media data provide relevant information for the analysis of socio-spatial behaviour patterns in urban environments. They allow mapping segmented variables and identifying points of interest—POIs—or areas of interest—AOIs—(Önder et al. 2014; García-Palomares et al. 2015; Leung et al. 2017; Maeda et al. 2018). Furthermore, thanks to the availability of dynamic georeferenced UGC and metadata context-evidence, LBSN data analysis is also useful to study imbalances, such as those deriving from overtourism. Examples include, among others, the integration of the so-called “Smart-City Lens”—considering “how human-technology interactions reframe the overtourism knowledge gaps” (Pasquinelli and Trunfio 2020)—; or the development of urban-destination marketing and management strategies (Qu and Zhang 2013).

This research sets out an innovative approach to describe and characterise urban tourist activities and to identify AOIs from a user perspective, using data from Instasights, Foursquare, and TripAdvisor as the main source. The results obtained help to understand the spatial distribution of tourist activity and can thus serve to balance functional asymmetries between areas, preventing phenomena such as overtourism or gentrification. The approach can also contribute to orientating land-use urban planning and the design of tourism policies in line with urban revitalisation and regeneration. The main novelty of the method is the ability to identify AOIs through LBSN data. Specifically, the three selected sources contribute differently to the aim of the study: instasights heatmaps serves to delimit an area spanning the most popular city locations (Avuxi Ldt 2022); Foursquare provides the POIs and check-in data of both, locals and visitors (Yang et al. 2018); and the TripAdvisor database informs about tourist interests regarding sites and activities (Van der Zee and Bertocchi 2018). Therefore, the tourists’ preferred venues could be identified and ranked, allowing to verify which urban areas were of interest to tourists and to guide the city’s development based on traces of user activity.

As acknowledged in previous studies, LBSN data represents an advantageous source of information to identify relevant places in the city. A number of straightforward methods have produced valid results, such as the identification of relevant *plazas* in Mediterranean cities (Martí et al. 2017). For example, based on this experience, the number of check-ins registered in Foursquare venues was used to identify relevant tourist POIs and then AOIs, thus verifying the validity of this method in a different context. Identifying tourist AOIs is an essential step to orientate tourism policies in terms of urban planning and management.

A case-study approach in the city of Bucharest, Romania was conducted to implement this exploratory study. The applied methodology allowed validating the suitability of the method to unveil urban tourism dynamics and to produce useful insights.

The remainder of this paper is structured as follows: Sect. 2 focuses on the literature review approaching the use of LBSNs to assess and identify POIs in tourist areas; Sect. 3 describes the context of the Bucharest case study; Sect. 4 presents the background of the sources and describes the proposed method; Sect. 5 presents the results; and finally, Sect. 6 includes a discussion of the findings and some concluding remarks.

## Literature review

The need to monitor the balance between tourism performance and urban liveability is particularly noteworthy in tourism research (Shoval and Ahas 2016; Martí et al. 2021). In this domain, it has been shown that the use of LBSN datasets helps to improve our understanding of urban phenomena (Bellini and Pasquinelli 2017). A comprehensive literature review on the application of big data to tourism has in fact been performed by Li et al. (2018) and by Salas-Olmedo et al. (2018).

Specifically, previous studies on POIs sourced by LBSNs have focused mainly on travel behaviour and tourist trajectories in order to provide custom recommendations using Foursquare (Massimo and Ricci 2019; Dietz et al. 2020; Stamatelatos et al. 2021) or TripAdvisor (Van der Zee and Bertocchi 2018), combined with other geolocated data from social networks, such as Twitter or Yelp. The characterisation in those studies, among many others, is oriented towards either improving tourist recommendation systems (Massimo and Ricci 2019; Stamatelatos et al. 2021) or identifying tourist destinations or traveller clusters (Dietz et al. 2019; Stavrakantonakis 2013). Despite the relevance of these topics (Li et al. 2018), contributions to the spatial behaviour of urban tourists in cities (Salas-Olmedo et al. 2018) are still lacking. Such spatial behaviour, however, represents a key factor that needs to be considered in the design of land-use urban policies and tourism management.

In line with this approach, pioneering research has made headway in the identification of Tourist Activity Centres, such as areas of interest in the cities of Valencia and Alicante, Spain (Martí et al. 2021)—based on Foursquare, Twitter, Google Places, and Airbnb. Progress has also been made in the use of LBSNs—Panoramio, Foursquare, and Twitter—to infer tourist activities from UGC in the case of Barcelona, Spain (Salas-Olmedo et al. 2018). By using geo-tagged data, these studies broaden our knowledge of tourist activity and reflect how the use of the land is highly complex, requiring an analysis not only from a tourist industry perspective but also from an urban planning perspective.

With respect to the methodology, McKenzie et al. (2015) developed a multi-dimensional characterisation of POI types via a Foursquare database. They demonstrated the validity of using this social network to provide temporal, thematic, and spatial distribution patterns within an urban context. The notion of areas of interest (AOIs) has emerged in addition to these POIs. AOIs integrate multiple scenic elements (Dennouni et al. 2018; Mai et al. 2018) and commonly agreed AOI features include: (1) the existence of a distinguishable core place related to a landmark—e.g., the ‘Central-Market’ area—; (2) the magnitude of its extension; or (3) its recognition as a whole. Such features, however, strongly vary according to users’ perceptions or experiences (Bennett and Agarwal 2007). Therefore, from a tourism management and urban planning perspective, it is of the utmost importance to recognise tourist AOIs to ensure a more accurate design of tourist and land-use urban policies. Considering the demonstrated advantages of LBSNs to identify POIs in cities, this study sets out a method to identify AOIs based on combining information from three LBSNs: Instasights heatmaps (Avuxi Ldt 2022), Foursquare (Foursquare Inc. 2019), and TripAdvisor (TripAdvisor LLC 2022). As explained next, all these platforms have been used in previous studies and have proven to be suitable baseline-data sources to address different tourism-related issues.

First, the Instasights heatmaps is an online tool—developed by Avuxi TopPlace heatmaps service—which provides an ‘Instant overview of the most popular areas within a city displayed in easy-to-understand map overlays’. It uses data on more than 200 million venues from over 70 public sources, updated daily (Avuxi Ltd 2022). Although this company’s specific market niche is online travel agencies and hotel metasearch sites, the website is also available to the public, offering informative maps throughout the world. Instasights heatmaps offer insights into different aspects of urban tourism phenomena, such as taking the pulse of urban dynamics following a public space renewal or identifying high concentrations of users within cities. Instasights heatmaps have provided relevant results when combined with: (1) Airbnb, to analyse the accommodation offer in tourist areas (Perez-Sanchez et al. 2018); (2) Foursquare, Google Places and Twitter, to develop a granular analysis of tourism-related places of interest (Martí et al. 2019a); or (3), TripAdvisor, to unveil the most photographed places (Simancas-Cruz et al. 2017).

Second, the check-in-based LBSN Foursquare includes a register of socio-economic activities and relevant places in the city, namely, venues (Williams and Chorley 2017; Foursquare Inc. 2019). Essentially, Foursquare users can share their location by ‘checking-in’ a given venue as well as their opinions and experiences about the specific place. The number of check-ins and visitors/users of each venue is accumulated as of the first time a venue is listed on the platform. This information has proven to be valuable to assess people’s preferences and perceptions of places (Van Canneyt et al. 2012; Martí et al. 2019a, b). Foursquare has been the data source of a broad range of studies covering topics such as: the analysis of performing trade areas (Zhang et al. 2013); the analysis of movement patterns and the popularity of urban areas (Silva et al. 2013); the study of traffic conditions (Izabel et al. 2014); the discovery of functional urban areas (Vaca et al. 2015); or the identification of the geographic distribution of venues across the cities (Çelikten et al. 2017). A number of works has also used Foursquare in combination with other LBSNs such as: (1) Google Places, to provide evidence of people’s activity and ranking of preferences (Martí et al. 2019a); (2) Twitter and Google Places, to uncover meaningful spaces through UGC (Martí et al. 2020); and (3) Panoramio, Foursquare and Twitter, to explore digital footprints of tourism and AOIs in Madrid, Spain (Salas-Olmedo et al. 2018). Foursquare has also been used in tourism studies in order to identify popular sites (Tammet et al. 2013) or activities related to tourism (Stavrakantonakis 2013).

Third, the TripAdvisor website is based on the idea that travellers rely on other travellers’ reviews to plan their trips. Currently, TripAdvisor has accumulated over 463 million single visitors and over a billion traveller reviews and opinions (TripAdvisor LLC 2021). This platform is one of the most recognisable consumer-generated-content sites, influencing many future travellers in their decisions (Simancas-Cruz et al. 2017). TripAdvisor, which was relaunched in 2018 as a social travel network that includes UGC, has recently gained attention as a source for research studies on customer behaviour and preferences, covering topics such as: market positioning effects (Martin-Fuentes et al. 2018, 2020); traveller decision-making (Litvin and Dowling 2018); or the influence of accessibility or walkability on tourists’ spatial behaviour (Hall and Ram 2019).

Overall, the reviewed literature has shown that the selected sources for the analysis of tourism dynamics—Instasights heatmaps, Foursquare and TripAdvisor—are valid and suitable sources of data, either as single sources or in combination with others. They contribute to a better understanding of human activity through user-generated data, including the possibility of discovering informal trends and providing a complementary approach to assess the use of urban space (Zhong et al. 2017).

## Bucharest as a case-study

Romania’s economy is mainly based on the service sector which accounts for 56% of the GDP and 48% of the workforce. Tourism, in particular, represents 5.3% of the GDP. Bucharest, the capital, emerged as a novel destination compared to other traditionally touristic cities such as Istanbul, Budapest, Prague, or Vienna (Iovitu et al. 2013) before the COVID-19 crisis. The situation will hopefully bounce back in the near future, continuing the previous trend. Evidence of Bucharest’s high potential as a tourist destination is its 283% increase in the number of tourists between 2015 and 2018, going from 635,000 visitors in 2015 to 1.8 million in 2018 (Alpopi et al. 2020). Despite the wonderful tourist attractions that have recently emerged in the city, the lack of a coherent official strategy to foster Bucharest as a tourist destination (Tigu et al. 2018; Surugiu et al. 2020) impedes the building of its own ‘image on the international tourism map’. Such a portrayal is frequently promoted as a side effect of airline marketing strategies, event celebrations, or ‘word of mouth’ (Tigu et al. 2018, p. 979). In this line, according to the recent research conducted by Camelia Surugiu et al. (2020), social media is a valuable tool to assess tourism services as it embodies a virtual, private, and voluntary promotion of the city. The analysis of Bucharest’s tourism realities based on social media data shows clear potentialities, making this city a suitable case study. Another favourable factor is Romania’s total number of social media users, which reached 12 million in January 2021. This number reflects a growth of 9.1% between 2020 and 2021, i.e., above the average of European countries (DataReportal 2021).

## Sources and method

Foursquare and TripAdvisor’s categories were matched in order to facilitate the selection of the preferred POIs and tourist activities in the area delimited by Instasights data in the city of Bucharest. Combining the data from these three sources—Instasights, Foursquare, and TripAdvisor—allows to offer comprehensive information and to ascertain the most-frequented tourist places, as well as characterise the types of related tourist activities.

**Fig. 1:**
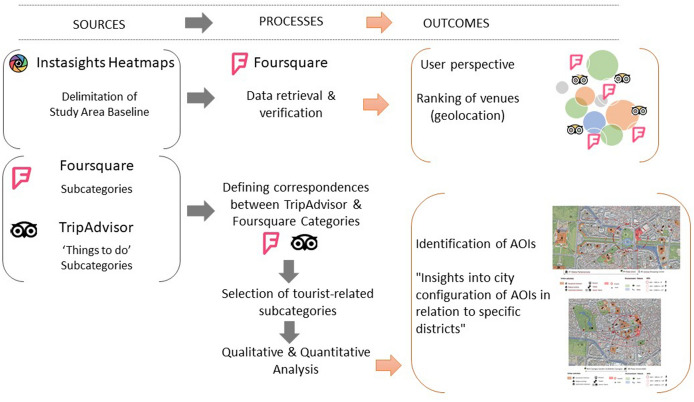
Main stages in the identification of AOIs via LBSN data categorisation

To summarise, the procedure included the following stages—see Fig. 1—: (1) the most popular tourist area was defined considering Instasights *Sightseeing* and *Shopping* vector-maps as baseline areas; (2) foursquare data contained in the delimited area were retrieved and verified (Martí et al. 2019b); (3) TripAdvisor categories and subcategories related to the ‘Things to Do’ grouping were scrutinised in order to identify the types of tourist activities that are ‘held on a permanent location and that offer travellers temporary things to do’ (TripAdvisor LLC 2022); (4) a correspondence between selected subcategories in TripAdvisor and Foursquare was established to find out which Foursquare registered venues could be considered to be related to tourism; (5) each selected venue was labelled following a new grouping system based on: general types of activities—label 1—; the nature of the activity, in terms of experience or service—label 2—; and the indoor or outdoor nature of the activity—label 3—; (6) to finish, the venues were mapped to interpret the results.

The interpretation of the results included the identification of AOIs via: (1) the statistical analysis of Local Moran’s I and, (2) the concentration of relevant venues according to the number of Foursquare users and check-ins.

### Description of data sources and their adequacy in this study

The three data sources provide comprehensible and accurate information on the location and characterisation of the tourist activity in the city.

**Fig. 2:**
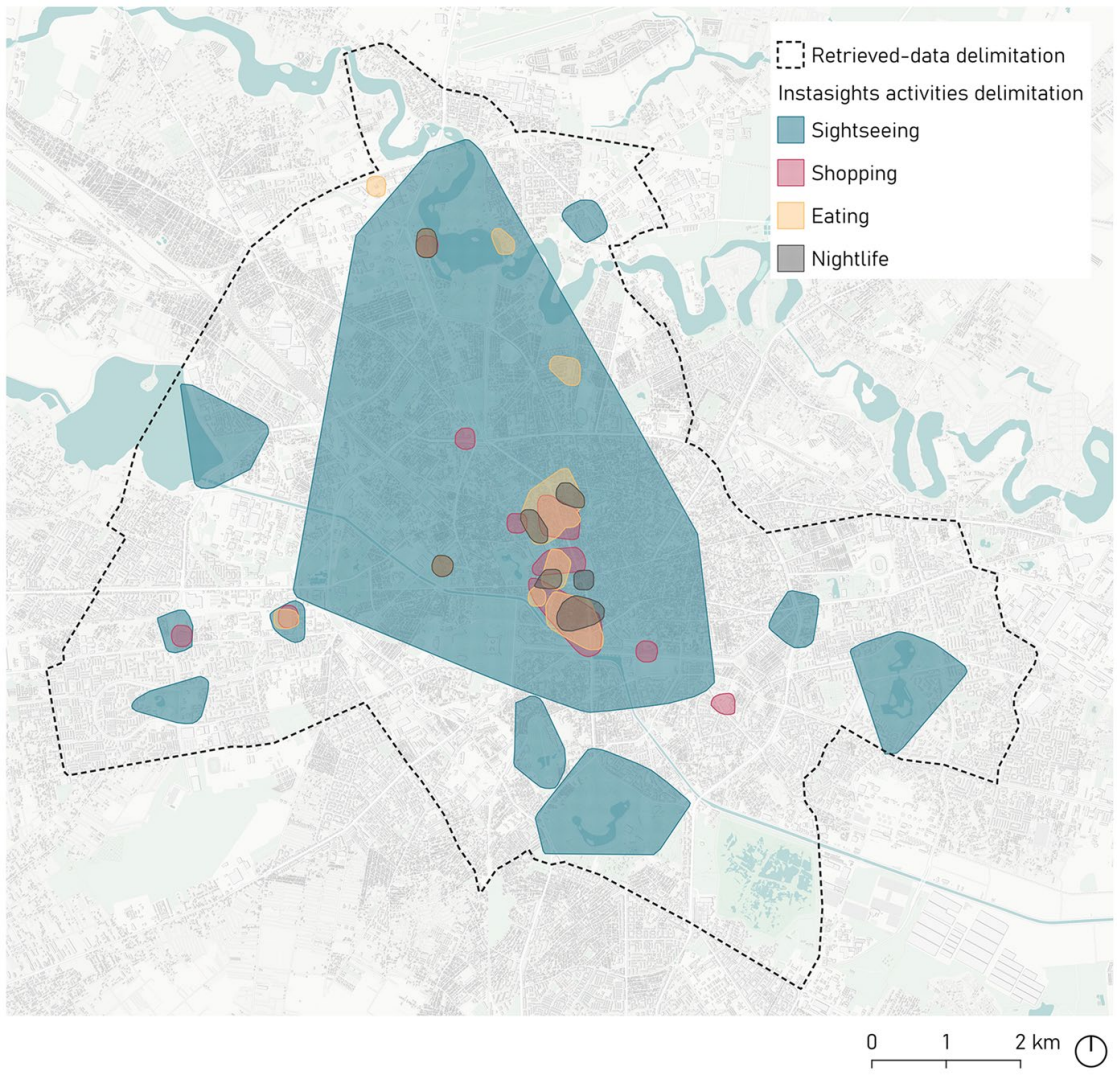
Delimited case-study area in Bucharest based on Instasights vector-maps relating to different tourist activity areas

First, Instasights heatmaps present the extension and density of four tourist activities—namely, *Shopping, Eating, Nightlife* and *Sightseeing—*as a five-level coloured gradient (Avuxi Ldt 2022). In this study, Instasights vector-maps were used as a baseline to define the scope of the study area enclosing the most relevant locations of tourist activity. The *Sightseeing* category spread across the broadest area in the city. A proper delimitation was thus defined by adjusting the Instasights limits to the nearest main urban axes—streets, avenues, boulevards. This final delimitation accounted for a surface area of 8163′6 ha and is presented in Fig. 2.

Second, Foursquare check-ins, i.e., users’ voluntary contributions, were used to gain a better understanding of which places in an urban area were popular (Silva et al. 2013). In this case, the general nature of its five-level venue categorisation hierarchy hindered the identification of tourist activity-related venues, even though some of the listed subcategories could directly evoke leisure or tourist activities—e.g., cruises, dive shops, flea markets, among others. Thus, Foursquare data can be considered biased regarding a city’s tourist hotspots since Foursquare users are both locals and tourists, indistinctly (Yang and Marmolejo Duarte 2019).

Third, regarding the information contained in TripAdvisor, the POI and activity listings were categorised according to their potential to meet the interests of tourists when visiting a city (TripAdvisor LLC 2022), in addition to the permanent places of interest to travellers. TripAdvisor listings were grouped according to the type of place or activity—*Restaurants, Things to do, Experiences, Accommodations, Vacation Rentals, Airlines*, and *Cruises*—and, in turn, into different categories linked to other more specific subcategories. In this case, we closely examined the general classification of categories and subcategories under the grouping ‘Things to do’. Indeed, only the permanent attractions or POIs located in a stable place—with an official name and a fixed address—were considered. Therefore, this selection met the criteria of the registered Foursquare venues and made it possible to match TripAdvisor subcategories with Foursquare subcategories. No other TripAdvisor grouping mentioned above was considered in the analysis—*Restaurants, Experiences, Accommodation, Vacation Rentals, Airlines*, or *Cruises*.

To summarise, Instasights provides delimitations of city areas which concentrate sightseeing activities. Foursquare provides a general listing of categorised venues that can be ranked by user preference, considering the highest number of registered check-ins. Additionally, TripAdvisor offers a categorised listing of tourist places, POIs, and activities. Thus, the retrieved Foursquare data of the city of Bucharest delimited by the Instasights *Sightseeing*-category vector-map ensured that the venues located inside these boundaries were potentially touristic. Moreover, the TripAdvisor categorisation of listings offers insights into the types of activities and places that potentially meet tourists’ interests. In this way, any Foursquare subcategory corresponding with that of TripAdvisor will contain the city’s preferred tourist venues. In turn, those venues can be ranked by user preference, reflecting the most popular places and activities in the city.

### Dataset information and processing

Bucharest city data sourced from Foursquare was retrieved using the Foursquare API service via a self-developed web application (Martí et al. 2019b), based on the area that had previously been delimited by Instasights heatmaps. The dataset was retrieved on 22 July 2019 and initially covered 21,605 venues, including the following venue-specific attributes: (1) venue name, (2) number of check-ins, (3) geographic coordinates (latitude and longitude) and (4), venue-related category and subcategory.

#### Data processing

The Foursquare dataset was verified in order to avoid any duplication and misrepresentation of venues (Martí et al. 2019b). Such data scrutiny increased the accuracy, generating a univocal identifier for each registered venue.

During the verification process, a total of 533 venues were discarded as they overlapped with others or were not categorised. The venues corresponding to the categories *Residence, Food, and Nightlife Spot* were discarded, either because they did not have any corresponding category in TripAdvisor—as in the case of *Residence*—, or the category was classified under another TripAdvisor grouping which was different from ‘Things to do’—such as *Food,* which corresponds to *Restaurants* in TripAdvisor. Therefore, once the filtering and verifying procedures had been conducted, the whole dataset listed a total of 16,522 venues, that were sorted into seven categories, namely: *Arts & Entertainment, College & Universities, Event, Outdoors & Recreation, Professional & Other Places, Shop & Services,* and *Travel & Transport*.

#### Selection of TripAdvisor subcategories and matching procedure

The next step was to work on the correspondence between subcategories, matching the Foursquare categories with similar ones in TripAdvisor—the results are presented in the supplementary material. To identify the Foursquare tourist-activity-related venues, TripAdvisor categories and subcategories included in the ‘Things to do’ grouping were examined in order to select those that included permanent attractions or POIs located in a specific place. An initial correspondence was established between TripAdvisor categories and subcategories and the Foursquare subcategories included in the dataset on the basis of their description (Foursquare Inc. 2019; TripAdvisor LLC 2022).

Moreover, to ensure accurate results in terms of the description of the tourist activity, an additional labelling task was performed. Specifically, further consideration was given to three issues via these labels: (1) the type of activity according to general groups—*Arts & Entertainment, Events, Recreation & Sports, Service, Shopping, Sightseeing & Landmarks, Travel & Transport, Wellness*—; (2) their nature—experience or service—; and (3), their spatial context—outdoors or indoors. Indeed, the Foursquare venues selected as tourist activities were labelled considering the nature of their corresponding subcategory; or, if no TripAdvisor subcategory corresponded to any other in Foursquare, the TripAdvisor listings in those subcategories were individually and manually identified in the Foursquare dataset and labelled on a case-by-case basis. Thus, to classify the venues, a data perusal was conducted together with a manual labelling, considering each subcategory or specific element features. Although they were initially classified as Foursquare venues, the matching of the Foursquare and TripAdvisor categories resulted in a more accurate and descriptive classification regarding tourism type and nature. Thus, all the selected venues fell under six Foursquare categories corresponding to 16 TripAdvisor categories. Their classification into groups, according to the proposed labels, facilitated the interpretation of the results in terms of the nature of the venues. Figure 2 shows the correspondence between TripAdvisor and Foursquare categories, including the proposed labels.

The criteria adopted to match and label the subcategories and the subsequent identification of tourist activities in the Foursquare dataset involved four types of actions:

1. When the TripAdvisor categories and subcategories had similar or identical names in the Foursquare classification, labels related to the type of activity, experience/service, and outdoors/indoors characterisation were assigned to all the venues included in the corresponding Foursquare subcategory.
2. When a Foursquare category or subcategory was identified as a potential tourist activity that could fall into a TripAdvisor category, labels were assigned to all the venues included in that corresponding Foursquare subcategory.
3. When the description of TripAdvisor categories and subcategories failed to match any Foursquare classification category, the names of the TripAdvisor listings were individually compared to all the venue names in the Foursquare dataset. They were manually labelled based on their specific features only in cases of consistency.
4. All Foursquare subcategories unrelated to tourist activity were discarded.

#### Data interpretation: analysis of selected venues

Once the tourist venues were identified, the selection was mapped following a twofold perspective. First, a general quantitative and qualitative analysis was performed based on the number and type of tourist venues according to the diverse categories. Second, AOIs were identified taking into account all the following factors: high concentration, functional diversity, tourist hotspot proximity, and relevant venues according to the number of Foursquare check-ins. Additionally, a Local Moran’s I bivariate analysis (Anselin 1995)—considering Foursquare venues and check-ins—allowed to assess whether the pattern of geolocated data expressed was clustered, dispersed, or random, and to determine statistical outliers according to specific types of activities and functionality. These results helped to define AOIs, including urban morphological conditions, such as venues facing the same public space or located within the same urban block. Finally, the venues and main relevant activities in an AOI were represented, thus allowing a thorough qualitative analysis that shed light on the particular features of the city’s tourist activity.

Specifically, it has been recognised that the standard European walking distance that users would be willing to cover was established in 500 m, the equivalent of a 9- to 10-min walk (Walker 2011; Richard Kuzmyak and Dill 2012). This measure, however, could not be strictly applied as tourists’ walking behaviours depend, among other factors, on urban multi-attraction (Yun et al. 2018; Caldeira and Kastenholz 2020). For this reason, additional walking routes covering longer distances were included in this study: 18 min, equivalent to 1000 m, and 27 min, equivalent to 1500 m.

The Local Moran’s I analysis is an inferential spatial statistic used to calculate local spatial autocorrelation. A positive I value implies that a variable has neighbouring characteristics with equally high or low attributes—cluster. A negative I value means that there are different neighbouring values—outliers.

The analysis output could be classified into five types of clusters: (1) High–High (HH): spatial clusters with high values, indicating a positive spatial autocorrelation—also called hotspots; (2) High–Low (HL): spatial clusters with high values adjacent to low values, indicating negative spatial autocorrelation—also called spatial outliers—; (3) Low–High (LH): spatial clusters with low values adjacent to high values, indicating a negative spatial autocorrelation —also called spatial outliers; (4) Low–Low (LL): spatial clusters with low values, indicating positive spatial autocorrelation—also called cold spots—; and (5) not significant: no spatial clusters between locations. The interpretation of the spatial analysis was based on the expected values, a pseudo p value, and a z score under the null hypothesis of no spatial autocorrelation—i.e., complete spatial randomness (Grekousis 2020).

The k-nearest neighbour method finds the k nearest observations for each observation of interest. In this case, we defined k = 4, corresponding to the 4 closest observations regardless of the distance between them. The number of neighbours (k) depends on the number of neighbour parameters. Thus, given the large number of neighbour values, 4 was the most appropriate number of neighbours to be included in the analysis.

## Results: identifying tourist activity through location-based social network information

The results are presented in two sections. First, we describe the identification of tourist activities and the description of their corresponding activity types. Second, we present the tourist activity patterns and characterisation in the case of Bucharest which were revealed through social media data.

### General analysis and description of results

**Fig. 3:**
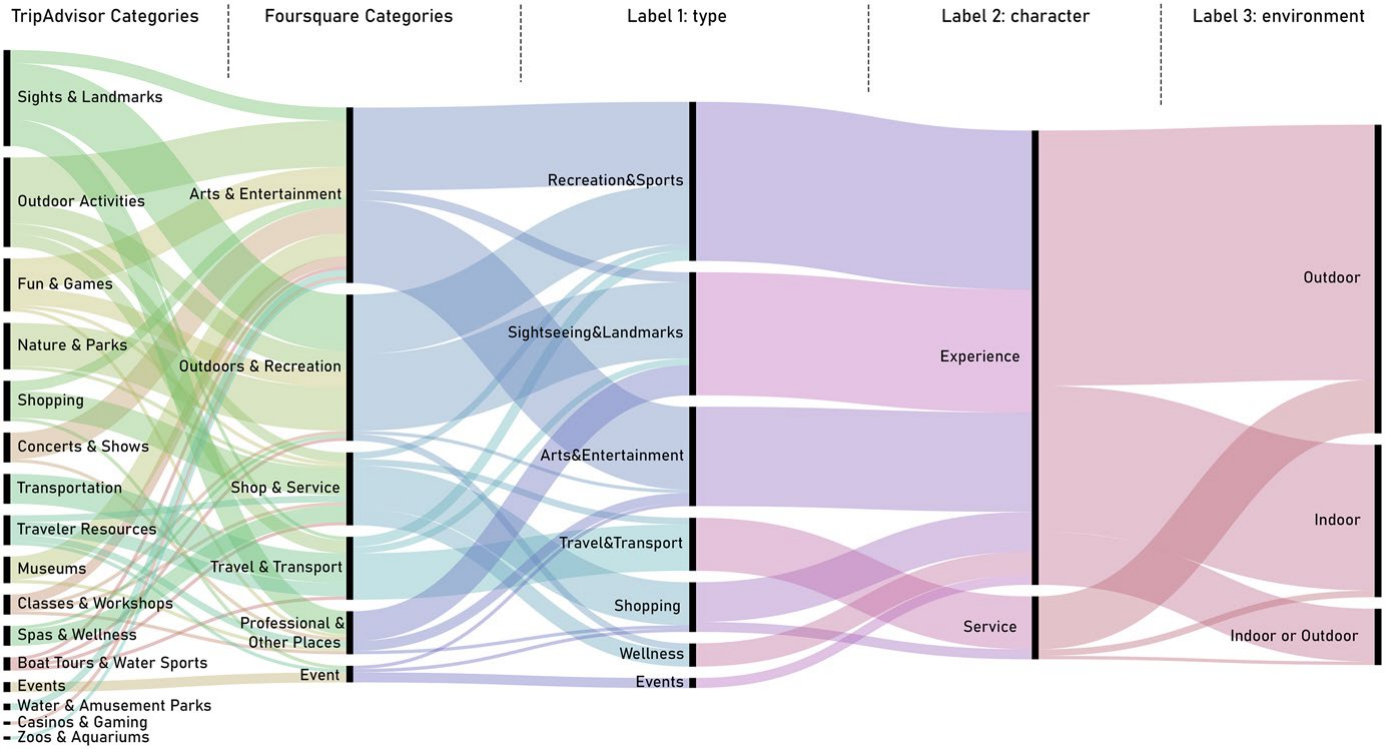
TripAdvisor categories corresponding to Foursquare categories and labels 1, 2, and 3

The matching of the TripAdvisor and Foursquare categories—Fig. 3—led to the selection of a total of 4421 venues from the whole dataset, accounting for 16,522 filtered venues. The selected venues comprised all the tourist activities within the established delimitation.

The classification into types of activities—Label 1—helped to explain the nature of the tourist activity and confirmed that tourist activities in Bucharest were mainly related to *Sightseeing & Landmarks*—39.5% of venues—, a widespread characteristic of urban tourism. *Arts & Entertainment*—19.9%—and *Recreation & Sports*—16.8%—were also relevant types of preferred tourist activities in the breakdown (Table 1). Despite this, very few *Wellness*, *Shopping*, or *Event* venues were considered of interest by tourists—6.2%, 4.3%, and 0.2%, respectively.

**Table 1:** Number of activities and representativeness (%) according to the type of activity—label 1

	Type of activity (label 1)	Number of activities	Percentage
1	Sightseeing and landmarks	1746	39.5
2	Arts and entertainment	878	19.9
3	Recreation and sports	743	16.8
4	Travel and transport	583	13.2
5	Wellness	273	6.2
6	Shopping	192	4.3
7	Events	8	0.2
	Total	4423	100.0

The *Travel & Transport* category accounted for a large share of the total venues—13.2%, see Table 1. Despite not being exclusively touristic, these venues contribute to the offer of tourist services. However, Tour providers, which could have been included as a *Travel & Transport* type of activity—Label 1—, were regarded as a *Recreation & Sports* activity, because the displacement service was considered part of the tourist experience.

**Fig. 4:**
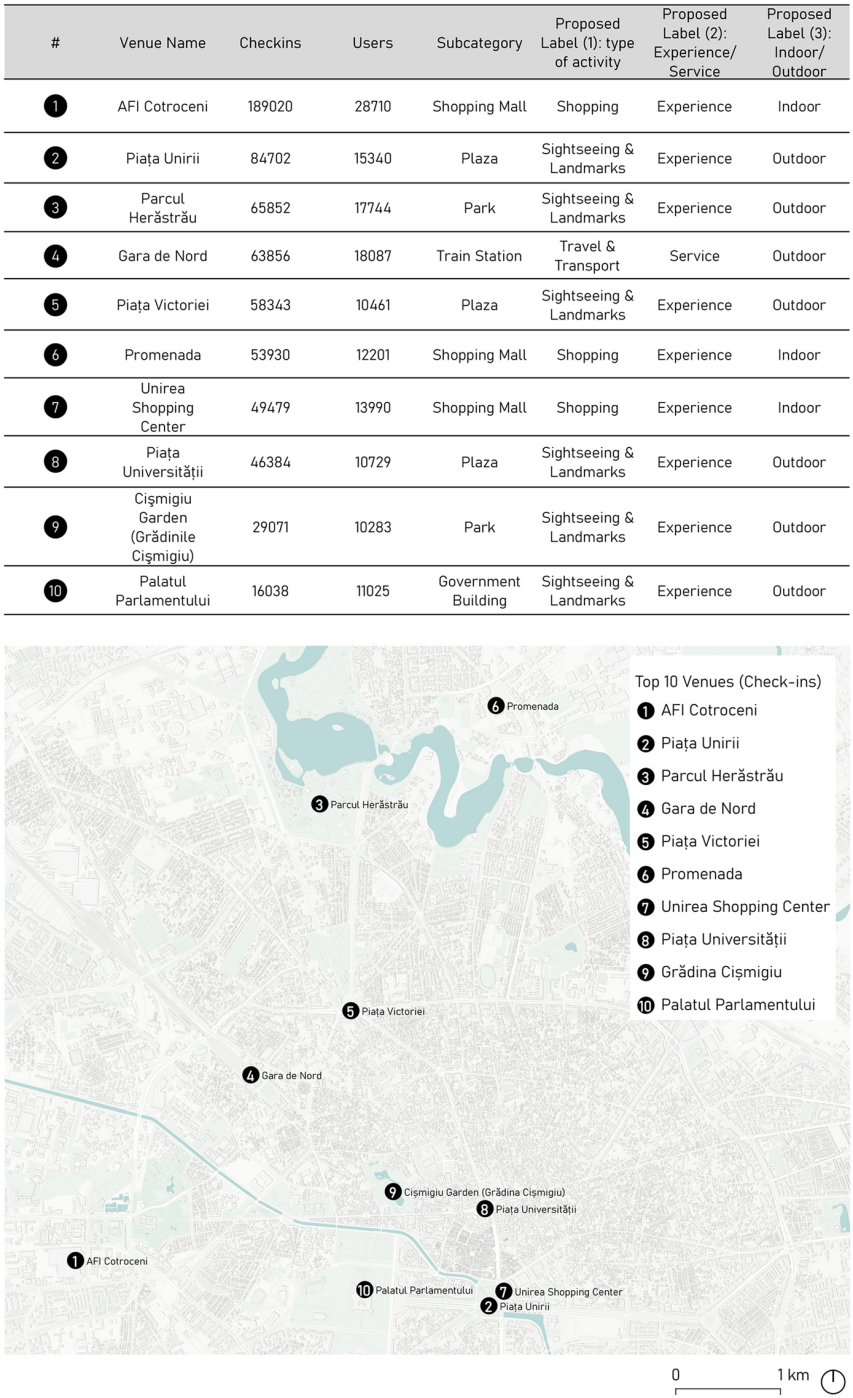
List and location of the top ten venues according to the Foursquare cumulative number of check-ins

The number of registered Foursquare check-ins per venue—as explained in Sect. 4.1—shed light on the top-ten venues (Fig. 4).

In relation to the *Experience* or *Service* classification of venues—Label 2—the results revealed that 86.1% of venues were experiential in nature—including a broad range of tourist attractions and activities, such as the Concert Hall Sala Palatului, or the historic site of Arenele Romane—while only 13.9% were regarded as tourist services. However, almost all services—94.8%—corresponded to *Travel & Transport* venues—such as bus stops or train stations—, while only 5.2% of the venues corresponded to other categories—such as Tourist Information centres or different types of establishments, including Currency Exchange shops.

In terms of the environment of the activities—Label 3—, the outdoor and indoor nature of the venues was balanced as the corresponding percentage was 54% and 46%, respectively. While outdoor activities were mainly related to categories such as *Sightseeing & Landmarks,* and *Travel & Transport*, indoor activities commonly included the venues related to *Shopping* and *Arts & Entertainment* categories. In this case, analysing the sample of the ten most checked-in venues, only the three shopping malls represented indoors activity, while the rest of the attractions were either public spaces or buildings that could be enjoyed from the outside, as in the case of the Governmental Building.

### Local Moran’s I analysis

**Fig. 5:**
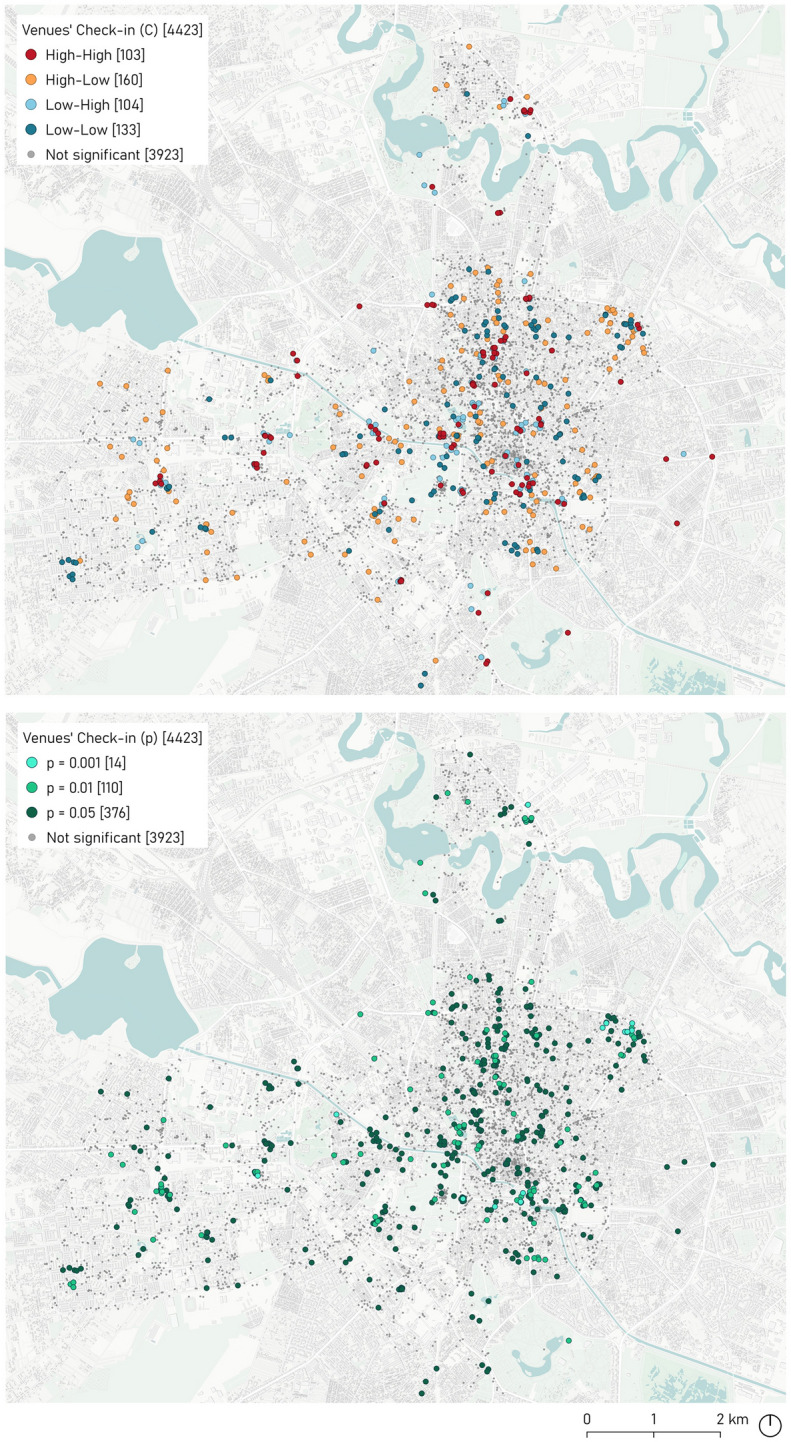
Local Moran’s I bivariate analysis based on Foursquare venues (N = 4423) for Bucharest

The analysis based on the Local Moran statistic was visualised in the form of a significance and cluster map—see Fig. 5. The spatial autocorrelation analysis functionality was rounded out by a range of operations to construct spatial weights using either boundary files,—contiguity based—, or point locations,—distance based. The neighbourhood or contiguity data set structure was formalised based on the spatial weights matrix (W), with elements wij = 0 when i and j were not neighbours, and non-zero, otherwise (Anselin et al. 2006). This analysis applied the k-nearest neighbour criterion which ensured that each observation had the same number of (k = 4) neighbours.

The spatial patterns of the tourist hotspot venues (HH and LL values) outline in a way how the city’s social life serves tourists’ preferences and shapes their choices. It also provides an understanding of the management and planning of the public spaces by the city administration. Hence, the results obtained allow to monitor the city functions: the high and low clusters were related to specific activities—Arts & Entertainment, Sightseeing & Landmarks, Wellness, Shopping, Travel & Transport—which emphasize tourists’ interests in particular types of activities.

The distribution of the spatial patterns differed according to the type of social activity and its location: the HH values were located near the tourist area of the city, mainly the city centre—that most tourists visit for its cultural attractions—and the LL values, which were near city locations of a more industrial nature where tourist services are less developed.

The bivariate analysis of Local Moran’s I outlines the relationship between the value of one variable at the location and the average of the neighbouring values of another variable (its spatial lag). It also measures the influence of one variable on the occurrence of another close variable. The results ranged between − 1 and 1, 0 implying no spatial autocorrelation. Significant local statistics meant the closer a value to 1, the greater the degree of positive spatial autocorrelation. In turn, the closer the value to − 1, the stronger the negative spatial autocorrelation. The inference of the analysis was based on a standardised Z-score (null hypothesis = spatial randomness).

Figure 5 shows the locations that presented significant local statistics. The degrees of significance are marked by increasingly dark shades of green. The High–High—HH—and Low–Low—LL—cluster locations are related to specific activities: *Recreation & Sports*, *Arts & Entertainment*, *Sightseeing & Landmarks*, *Wellness*, *Shopping*, *Travel & Transport*. The experience of locals and visitors encompassed both indoor and outdoor types of locations—Fig. 5.

The spatial autocorrelation given by Moran’s I produced the top ten hotspot list of venues—AFI Cotroceni Mall, Unirii Square, Romana Square, Herastrau Park, North Station, Victoriei Square, Promenada, Unirea Shopping Center, Plaza Romania Mall—and, additionally, four clusters with a high number of check-ins—AFI Cotroceni Mall, Victoriei Square, Unirea Shopping Centre and Plaza Romania Mall.

The high value clusters—HH—were in central areas where most tourist accommodations and attractions were located—near Banat Hotel, Radison Hotel, Lido Hotel, Euro Kogalniceanu Hotel—, as well as key transport facilities—the metro station near Bucharest University, the railway station—revealing a *touristification* with an emphasis on the city centre (van der Zee et al. 2018). The HH locations tended to cluster in different functional areas of the city specialised in *Sightseeing & Landmarks* and *Shopping*, usually situated near tourist attractions and different *Travel & Transport* facilities.

The low-value clusters—LL—were located near the city centre neighbouring the business centre—Parlamentului Palace. The concentration of tourist activities and services made it possible to distinguish the tourist areas in the historic city centre. However, the industrial and business institutions located near tourist attractions or accommodation represented the outliers.

The findings outline the popularity attributed to hotspot venues, based on the social activities chosen by tourists in Bucharest. The analysis revealed hotspot activities in different squares—Working Square, Buzesti Square—, in various parks—Carol Park, Union park—, historical sites—Arenele Romane—, the Cismigiu lake, and government buildings such as Victoria palace.

### Identification of AOIs in the city of Bucharest through Foursquare data and qualitative interpretation of results

**Fig. 6:**
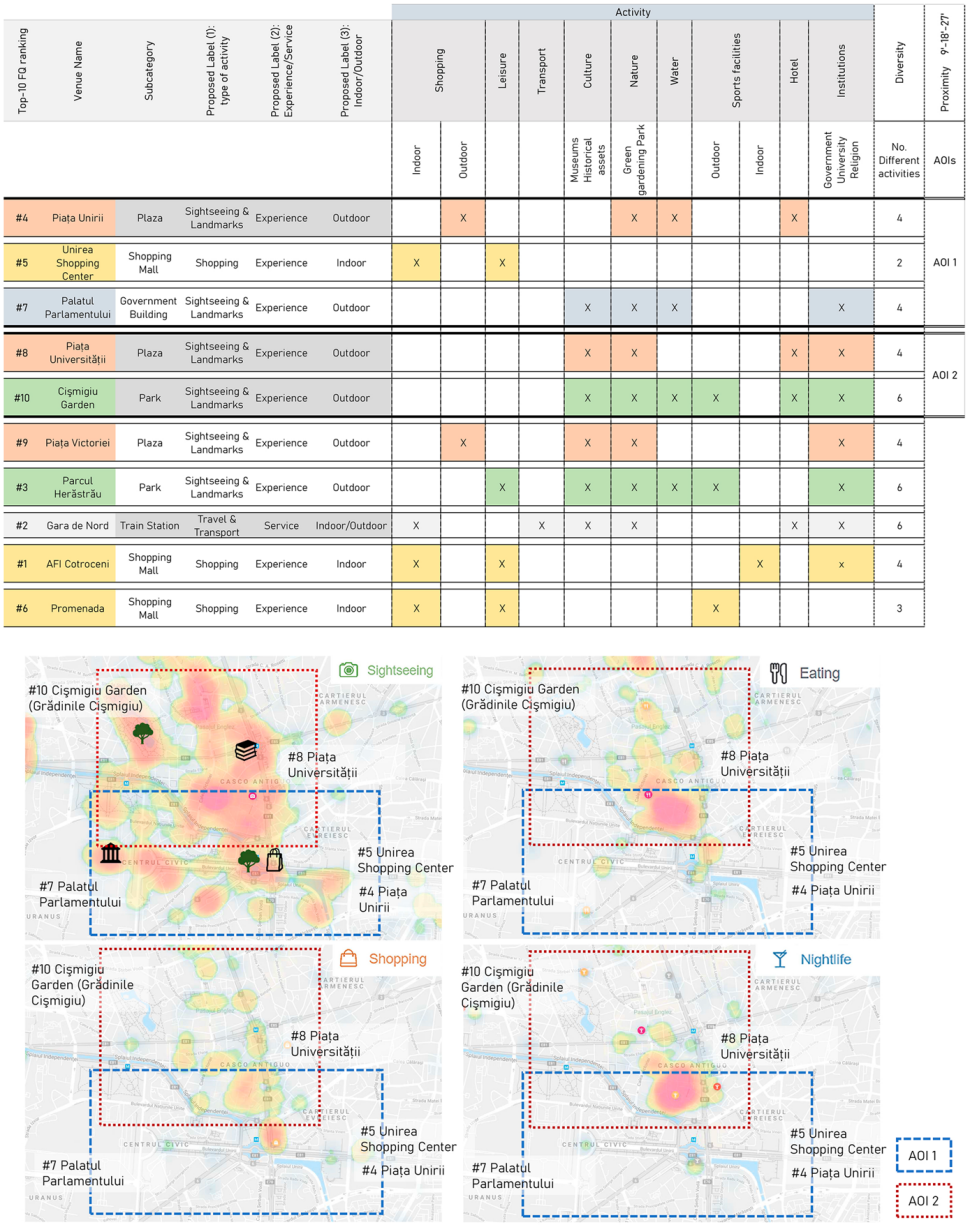
Summary of the data for the identification of AOIs and general delimitations on Bucharest’s Instasights thematic heatmaps—*Sightseeing, Eating, Shopping* and *Nightlife*

In this study, AOIs were defined by combining the following criteria: (a) existence of POIs included in the top-ten-venue ranking—Fig. 6; (b) proximity between different POIs within three estimated walking-time slots represented as average walking distances—9 min or 500 m, 18 min or 1000 m, and 27 min or 1500 m—; and (c) the presence of a variety of activities which contribute to urban dynamism linked to tourist interests such as cultural assets, leisure activities or shopping, among others. Based on these criteria, Fig. 5 summarises the analysis of the top ten venues. The order of the venues was consistent with venue proximity and helped to identify synergies between the venues that could function together as a system of attraction of tourist activity.

**Fig. 7:**
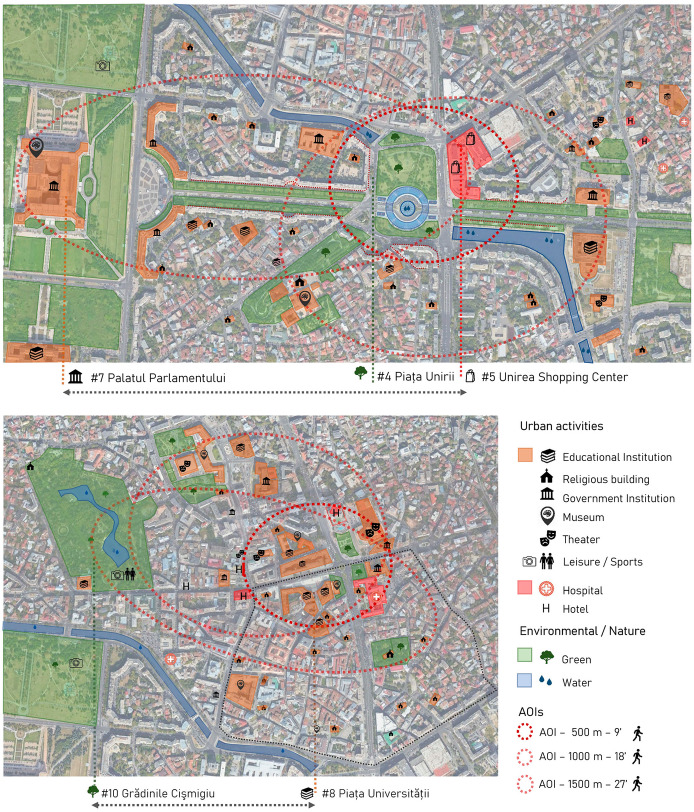
AOI 1—Plaza Unirii—Unirea Shopping Center—Unirii Bulevard—Palatul Parlamentului—. AOI 2—Piața Universității-Cişmigiu Garden (Grădinile Cişmigiu)

Under these conditions, two AOIs emerged. First, Area of interest 1—AOI 1—gathered three main venues—Fig. 7—, two of which, Piața Unirii and Unirea Shopping Centre, came together and constituted the same urban space with complementary outdoor and indoor activities. The third venue was the Palatul Parlamentului, which was slightly further away but within the 1500 m distance. Second, Area of interest 2—AOI 2—interrelated Piața Universității and Cişmigiu Garden within a distance of 1000 m—Fig. 7.

When analysed in detail, each AOI offered a different urban experience to visitors. Starting with AOI 1—Fig. 7—, the central node—500 m or a 10-min walk—includes Piața Unirii and the Unirea Shopping Centre. These venues collected 134,181 Foursquare check-ins, and both were part of an urban whole offering six complementary activities, providing dynamism and complexity to the urban scene. From this perspective, water and green areas were spotlights in a public space’s monument area, connecting the urban tissue in a multiscale approach. Looking at the middle distance, i.e., within 1000 m, and scattered around the urban tissue, one can observe numerous institutional, educational, and religious buildings. Lastly, within the 1500 m walking distance, the Bulevardul Unirii, full of greenery and presenting a monumental scale, is the main axis connecting Piața Unirii to Palatul Parlamentului, another relevant tourist POI. This latter venue, the most distant one within AOI 1, incorporates two new types of activities: a cultural activity—a museum—and an institutional one—a governmental body—that provide additional complexity and dynamism to the AOI 1.

Continuing with AOI 2—Fig. 7—, Piaţa Universităţii, with 46,384 Foursquare check-ins, is the central node within 500 m and belongs to Bucharest’s historical centre. The area is characterised by denser types of buildings, and a more human scale than AOI 1, with a smaller public green surface area. A large number of educational and institutional venues which cannot be considered specifically as tourist activities also emerged nevertheless as urban sightseeing hotspots and were located at short and middle distances. Finally, within the middle-long 1000–1500 m distance range, the location of one of the big urban parks, the Cişmigiu Garden, offered six different types of activities linked to touristic or visitor interests. It is surrounded by several urban itineraries with a higher concentration of restaurants and retail, providing a more dynamic public scene, as reflected in the Instasights heatmaps in Fig. 5.

In short, based on functional diversity and different types of walking routes, it was possible to identify tourist activity AOIs at the urban level by using UGC from LBSNs.

## Discussion and conclusions

This work demonstrates that the identification of tourist activities is not as simple as it may seem, despite the substantial number and range of tourist activities that exist in conventional cities compared to other economic activities. Martí et al. (2017) proposed an innovative and reliable approach to determine relevant places in a city. The most remarkable result to emerge from our work, however, was that the method used to identify relevant tourist places/areas is not such a straightforward procedure.

The proposed method is focused on the analysis of the city’s concentration of tourist activities that provides a better understanding of the urban functional complexity. Despite additional information on the users’ origin—locals or visitors—would be truly helpful to incorporate nuances into the interpretation of the results when analysing tourist activity, the proposed method presents accurate results for identifying tourist hotspots and activity nodes. In short, the accumulated number of check-ins over time indicates overall people’s preferences over places and, filtering the Foursquare venues by using the list of tourist activities in TripAdvisor, offers without any doubt which are the tourism-related areas of interest.

In this work, the main advantages of the proposed methodological approach are threefold. First, the three selected sources effectively fulfil their mission and consider user perspective as a means to: (a) identify the study area baseline, which defines the scope of the data retrieval; (b) provide a ranking of formal and informal places based on users’ preferences—locals and visitors—by using the Foursquare dataset; and (c) provide a clear selection of tourist activities. Second, social media data are considered a cost-efficient source to rapidly monitor a broad range of issues compared to both governmental institution databases and the traditional field techniques of data collection. Third, the matching of Foursquare and TripAdvisor tourist activity subcategories allowed combining these LBSN data, reflecting a rising complexity, and offering additional nuances to the conducted analysis.

The methodology proved to be a complementary approach, providing a focus for tourism management and urban planning strategies. It combined appropriate sources for the recognition of tourist AOIs in the city. In addition, the matching of data between Foursquare—a user-generated database with geolocated information—and TripAdvisor—a curated list of tourist information—proved to be a useful and timely method to identify AOIs based on tourist venue significance from a user perspective. Moreover, this method generated insights into the city’s AOI configuration in relation to specific districts or neighbourhoods.

In line with previous studies, the present work responded to the conclusion of Salas-Olmedo et al. (2018) and Martí et al. (2021) according to whom several sources should be used in a complementary manner when considering LBSNs as data sources to analyse a city’s tourist activity. Moreover, the study contributes to the discussion on the need to address AOIs. The sole identification of POIs or landmarks fails to account for a city’s complex tourist activity which encompasses very different types of economic activities. Thus, the proposed criteria in terms of POI proximity, diversity, and interest proved to be effective to determine AOIs according to the conditions described by Bennett and Agarwal (2007). In our case, the selected ‘Things to do’ venues were sufficient to obtain accurate information on the characterisation of AOIs, considering that neither the accommodation nor the catering sector were addressed in this study. Incorporating data from these two complex and relevant sectors for tourist activity would contribute to building a more intricate approach. This issue could be addressed in further research.

A general method, resembling that proposed here, could be designed considering the TripAdvisor and Foursquare general classification of categories and subcategories. However, a perusal of the data would be necessary in each case to ensure accuracy and to avoid venue classification misinterpretations. This fact, therefore, limited the obtention of immediate results. Moreover, further results were provided based on the labelling of the venues in terms of: (1) types of activities; (2) the nature of the activity as an experience or service; and (3), their indoor or outdoor nature. This task needs to be conducted manually. Another limitation is the availability of a representative amount of Foursquare data given that the use of Foursquare is not as widespread as that of other networks. An alternative source, such as the Google Places ranking, based on the starred locations, could be used to infer users’ venue preferences due to its larger and more homogeneous penetration rate. However, to the best of our knowledge, this source has not yet been used for this purpose, and could be considered in future studies on the topic.

Specifically, the Bucharest study produced interesting findings in relation to the POIs’ functional diversity and the delimitation of specific AOIs.

First, relevant tourist AOIs emerged from the identification of users’ interests and experiences, providing a novel approach that can be useful in the design of future tourism strategies as well as city urban planning. This "people-based" perspective, through virtual traces of user behaviour, preferences, activities and perceptions, provides tourism management and urban planning with the opportunity to broaden their knowledge of a city’s functioning, which in turn, allows addressing issues such as: (a) the identification and delimitation of "inactive areas"—urban activity voids—that may require a local urban renewal plan; (b) the detection of hidden popular spots that can trigger urban regeneration processes, spurring urban vitality by aligning relevant tourist activities with the habits of local residents.

Second, the detected AOIs included several institutional, academic, and administrative buildings whose programmes were not specifically related to tourism. Most presented an interesting monument and heritage combination which increased tourist attractiveness. They did not, however, offer any particular tourist experience. Conversely, shopping centres and parks were susceptible of being revisited because they offer experiential immersion. Therefore, a thorough understanding of functional diversity and the location of these functions contributes to a better understanding of the imbalances in the spatial distribution of tourist activity. These imbalances can be observed at a multi-scale and multi-functional level.

On the one hand, at the city level, concentration patterns of preferred activities can reveal functional discontinuities. In these cases, urban planning or tourism policies could boost city vitality by strategically linking active fragments that can complete a coherent city’s functional structure. Additionally, the proposed method allows linking traditionally acknowledged POIs with other areas that informally emerge from users' interests and habits.

On the other hand, at the local level, specific and more accurate action plans could be developed by incorporating comprehensive data processing. A thorough analysis of the diversity of urban and economic activities in combination with the interests of users, whether locals or visitors, could support the creation of specific activities to enhance urban vitality in certain itineraries or clusters. The implementation of promotional actions—from the tourist management perspective—in less highly recognised tourist trails would improve their potential as urban attractors. In addition, strategic locations could be identified to locate park-and-ride facilities, including visitor bus stops or rent-a-car businesses, that would complete a sustainable mobility offer by reducing traffic pressure in the busiest areas.

Furthermore, overtourism, which was undoubtedly a central tourism management problem prior to the COVID-19 pandemic, could be analysed using the methodological approach developed in this study. Indeed, it showed its potential as a monitoring tool for land-use urban planning for this type of functional imbalance in tourist activity. Although overtourism did not represent a specific problem in the case of Bucharest at the time of the study, many European capital cities have been negatively affected by this phenomenon. This is why further research could be conducted on this topic by applying the proposed method. Tourist functions could be monitored during the recovery period in areas that previously have recognised as suffering from overtourism.

Finally, the present study opens two lines of research. The first is the exploration of whether this method allows to appropriately depict the city’s informal tourism dynamics. In other words, if it allows identifying not only the most well-known tourist hotspots but also unexpected popular areas. The second is the exploitation of UGC to identify emerging spatial imbalances due to a lack of attractiveness and continuity within the city’s overall distribution of activities. Moreover, future studies may have to address the effects of current COVID-19 restrictive measures that could affect tourist behaviours in terms of activities and places in the city. Therefore, the use of location-based social media data presents a huge potential as a data source in future urban analyses, policy-making and urban planning.

## Supplementary Information

Below is the link to the electronic supplementary material.

Supplementary file1 (PDF 9922 kb)

## Data Availability

The data that support the findings of this study are available from the corresponding author, upon reasonable request. Restrictions apply to the availability of the raw data from Foursquare.

## References

[CR1] Alpopi C, Diaconu S, Velicu ER (2020). Strategies on the development of ecotourism at the Bucharest in the context of globalization. SHS Web Conf.

[CR3] Anselin L (1995). Local indicators of spatial association—LISA. Geogr Anal.

[CR4] Anselin L, Syabri I, Kho Y (2006). GeoDa: an introduction to spatial data analysis. Geograph Anal.

[CR5] Arribas-Bel D (2014). Accidental, open and everywhere: emerging data sources for the understanding of cities. Appl Geogr.

[CR6] Avuxi Ldt (2022) InstaSights. Avuxi top place heatmaps. https://www.avuxi.com/topplace Accessed 10 Jan 2022

[CR7] Bellini N, Pasquinelli C, Bellini N, Pasquinelly C (2017). Urban tourism and city development: notes for an integrated policy agenda. Tourism in the city: towards an integrative agenda on urban tourism.

[CR8] Bennett B, Agarwal P (2007) Semantic categories underlying the meaning of place. En lecture notes in computer science (including subseries lecture notes in artificial intelligence and lecture notes in bioinformatics) (vol 4736 LNCS), pp 78–95. 10.1007/978-3-540-74788-8_6

[CR9] Boivin M, Tanguay GA (2019). Analysis of the determinants of urban tourism attractiveness: the case of Québec City and Bordeaux. J Destin Market Manag.

[CR10] Caldeira AM, Kastenholz E (2020). Spatiotemporal tourist behaviour in urban destinations: a framework of analysis. Tour Geogr.

[CR11] Çelikten E, Falher GL, Mathioudakis M (2017) Modeling urban behavior by mining geotagged social data. In: IEEE transactions on Big Data. pp 220–233. 10.1109/TBDATA.2016.2628398

[CR12] Cheng Z, Caverlee J, Lee K, Sui DZ (2011) Exploring millions of footprints in location sharing services. In: ICWSM (vol 2010). pp 81–88

[CR13] DataReportal (2021) Digital 2021: Romania—Global digital insights. https://datareportal.com/reports/digital-2021-romania. Accessed 2 May 2022

[CR14] Dennouni N, Peter Y, Lancieri L, Slama Z (2018). Towards an incremental recommendation of POIs for mobile tourists without profiles. Int J Intell Syst Appl.

[CR15] Dietz LW, Roy R, Wörndl W (2019). Characterisation of traveller types using check-in data from location-based social networks. Inf Commun Technol Tour.

[CR16] Dietz LW, Sen A, Roy R, Wörndl W (2020). Mining trips from location-based social networks for clustering travelers and destinations. Inf Technol Tour.

[CR17] Foursquare Inc. (2019) Foursquare venue categories. https://developer.foursquare.com/docs/resources/categories. Accessed 18 Feb 2021

[CR18] García-Palomares JC, Gutiérrez J, Mínguez C (2015). Identification of tourist hotspots based on social networks: a comparative analysis of European metropolises using photo-sharing services and GIS. Appl Geogr.

[CR19] Grekousis G (2020). Spatial analysis methods and practices describe. Explore—explain—though GIS.

[CR20] Hall CM, Ram Y (2019). Measuring the relationship between tourism and walkability? Walk Score and English tourist attractions. J Sustain Tour.

[CR21] Hernández JM, Kirilenko AP, Stepchenkova S (2018). Network approach to tourist segmentation via user generated content. Ann Tour Res.

[CR22] Iovitu M, Radulescu C, Dociu M (2013). Tourism planning in urban areas—trends, best practices and priorities in Bucharest. J Knowl Manag Econ Inf Technol.

[CR23] Izabel A, Tostes JS, Thiago H, Duarte-Figueiredo, F, Loureiro AAF (2014) Studying traffic conditions by analyzing foursquare and instagram data. En PE-WASUN '14: 11th ACM symposium on performance evaluation of wireless ad hoc, sensor, and ubiquitous networks, pp 17–24

[CR24] Kemp S (2020) Digital 2020: global digital overview—DataReportal—global digital insights. https://alpha.globalwebindex.com/. Accessed 2 May 2022

[CR25] Krehl A, Siedentop S, Taubenböck H, Wurm M (2016). A comprehensive view on urban spatial structure: urban density patterns of German city regions. ISPRS Int J Geoinf.

[CR26] Leung R, Vu HQ, Rong J (2017). Understanding tourists' photo sharing and visit pattern at non-first tier attractions via geotagged photos. Inf Technol Tour.

[CR27] Li J, Xu L, Tang L, Wang S, Li L (2018). Big data in tourism research: a literature review. Tour Manag.

[CR28] Litvin SW, Dowling KM (2018). TripAdvisor and hotel consumer brand loyalty. Curr Issue Tour.

[CR29] Maeda TN, Yoshida M, Toriumi F, Ohashi H (2018). Extraction of tourist destinations and comparative analysis of preferences between foreign tourists and domestic tourists on the basis of geotagged social media data. ISPRS Int J Geoinf.

[CR30] Mai G, Janowicz K, Hu Y, Gao S, Zhu R, Yan B, Ragalia B (2018) Collections of points of interest: how to name them and why it matters. In: Martin R, Shaowen W, Mengyo G, David J, Peter K (Eds) Spatial big data and machine learning in GIScience. Workshop at GIScience 2018. Melbourne, Leibniz-Zentrum für Informatik, Germany, Australia, pp 29–54

[CR31] Martí P, Serrano-Estrada L, Nolasco-Cirugeda A (2017). Using locative social media and urban cartographies to identify and locate successful urban plazas. Cities.

[CR32] Martí P, García-Mayor C, Serrano-Estrada L (2019). Identifying opportunity places for urban regeneration through LBSNs. Cities.

[CR33] Martí P, Serrano-Estrada L, Nolasco-Cirugeda A (2019). Social media data: challenges, opportunities and limitations in urban studies. Comput Environ Urban Syst.

[CR34] Martí P, García-Mayor C, Serrano-Estrada L (2021). Taking the urban tourist activity pulse through digital footprints. Curr Issue Tour.

[CR35] Martí P, García-Mayor C, Nolasco-Cirugeda A, Serrano-Estrada L (2020) Green infrastructure planning: unveiling meaningful spaces through Foursquare users' preferences. Land Use Policy 97(March). 10.1016/j.landusepol.2020.104641

[CR36] Martin-Fuentes E, Mateu C, Fernandez C (2018). Does verifying uses influence rankings? Analyzing Booking.Com and Tripadvisor. Tour Anal.

[CR37] Martin-Fuentes E, Mateu C, Fernandez C (2020). The more the merrier? Number of reviews versus score on TripAdvisor and Booking.com. Int J Hosp Tour Adm.

[CR38] Massimo D, Ricci F (2019). Clustering users’ POIs visit trajectories for next-POI recommendation. Inf Commun Technol Tour.

[CR39] McKenzie G, Janowicz K, Gao S (2015). POI pulse: a multi-granular, semantic signature-based information observatory for the interactive visualization of big geosocial data. Cartographica.

[CR40] Miguéns J, Baggio R, Costa C (2008). Social media and tourism destinations: TripAdvisor case study. Adv Tour Res.

[CR41] Önder I, Koerbitz W, Hubmann-Haidvogel A (2014). Tracing tourists by their digital footprints: the case of Austria. J Travel Res.

[CR42] Önder I, Wöber K, Zekan B (2018) ECM benchmarking report—European cities marketing. https://www.europeancitiesmarketing.com/ecm-benchmarking-report/. Accessed 2 May 2022

[CR43] Pasquinelli C, Trunfio M (2020). Reframing urban overtourism through the Smart-City Lens. Cities.

[CR44] Perez-Sanchez V, Serrano-Estrada L, Marti P, Mora-Garcia R-T (2018). The what, where, and why of Airbnb price determinants. Sustainability.

[CR45] Qu Y, Zhang J (2013) Trade area analysis using user generated mobile location data. En WWW '13: 22nd international conference on World Wide Web, pp 1053–1064. 10.1145/2488388.2488480

[CR46] Richard Kuzmyak J, Dill J (2012). Walking and bicycling in the United States: the who, what where, and why. TR News.

[CR47] Salas-Olmedo MH, Moya-Gómez B, García-Palomares JC, Gutiérrez J (2018). Tourists' digital footprint in cities: comparing big data sources. Tour Manag.

[CR48] Shoval N, Ahas R (2016). The use of tracking technologies in tourism research: the first decade. Tour Geogr.

[CR49] Silva TH, Horizonte B, Salles J, Loureiro AAF (2013) Urban social behavior. A comparison of Foursquare and Instagram to the study of city dynamics and urban social behavior. En UrbComp '13. Chicago. 10.1145/2505821.2505836

[CR50] Simancas-Cruz M, Peñarrubia-Zaragoza MP, Rodríguez-Darias AJ, Padrón-Ávila H, Padrón-Candelario E, Suárez-Perera D (2017) La toma inteligente de decisiones en los procesos de planificación de destinos turísticos de litoral: el plan de infraestructuras turísticas de canarias (PITCAN). In: Fernando Vera-Rebollo J, Ivars-Baidal JA, Celdrán Bernabeu MA (eds) Actas del Seminario Internacional Destinos Turísticos Inteligentes: nuevos horizontes en la investigación y gestión del turismo. pp 143–166. 10.14198/Destinos-Turisticos-Inteligentes.2017.07

[CR51] Stamatelatos G, Drosatos G, Gyftopoulos S (2021). Point-of-interest lists and their potential in recommendation systems. Inf Technol Tour.

[CR52] Stavrakantonakis I (2013). Personal data and user modelling in tourism. Inf Commun Technol Tour.

[CR53] Surugiu C, Mazilescu R, Tudorache D-M, Astefanoaiei R, Surugiu M-R (2020) Is social media a valuable tool for evaluation of tourism services? Evidences for Bucharest Metropolis as a Tourism. Revista de turismo. Studii si cercetari in turism (29)

[CR54] Tammet T, Luberg A, Järv P (2013). Sightsmap: crowd-sourced popularity of the world places. Inf Commun Technol Tour.

[CR55] Tigu G, Sanchez AG, Stoenescu C, Gheorghe C, Sabou GC (2018). The destination experience through Stopover tourism—Bucharest case study. Amfiteatru Econ.

[CR56] TripAdvisor LLC (2021) Investor relations. https://ir.tripadvisor.com/investor-relations. Accessed 8 Dec 2021

[CR57] TripAdvisor LLC (2022) Things to do. Listing guidelines and categories. https://www.tripadvisor.com/Trust-lvz7RK0Xs9UI-Listings_policies_and_guidelines.html. Accessed 12 Jan 2022

[CR58] UNWTO—World Tourism Barometer (2022) vol 20 (2). 10.18111/wtobarometeresp.2022.20.1.2. Accessed 04 May 2022

[CR59] Vaca C, Quercia D, Bonchi F, Fraternali P (2015) Taxonomy-based discovery and annotation of functional areas in the City. En ninth international AAAI conference on web and social media. pp 445–453

[CR60] Van Canneyt S, Van Laere O, Schockaert S, Dhoedt B (2012) Using social media to find places of interest: a case study. GEOCROWD 2012—proceedings of the 1st ACM SIGSPATIAL international workshop on crowdsourced and volunteered geographic information. 10.1145/2442952.2442954

[CR61] Van der Zee E, Bertocchi D (2018). Finding patterns in urban tourist behaviour: a social network analysis approach based on TripAdvisor reviews. Inf Technol Tour.

[CR62] Van der Zee E, Bertocchi D, Vanneste D (2018). Distribution of tourists within urban heritage destinations: a hot spot/cold spot analysis of TripAdvisor data as support for destination management. Curr Issue Tour.

[CR63] Van Meeteren M, Poorthuis A (2018). Christaller and ‘big data’: recalibrating central place theory via the geoweb. Urban Geogr.

[CR64] Walker J (2011) Basics: walking distance to transit—human transit. https://humantransit.org/2011/04/basics-walking-distance-to-transit.html. Accessed 13 May 2021

[CR65] We are social (2022) Digital yearbook 2022. https://www.slideshare.net/DataReportal/digital-2022-global-overview-report-january-2022-v05. Accessed 5 May 2022

[CR66] Williams MJ, Chorley MJ, Sloan L (2017). Foursquare. The SAGE handbook of social media research methods.

[CR67] Xu F, Nash N, Whitmarsh L (2019). Big data or small data? A methodological review of sustainable tourism. J Sustain Tour.

[CR68] Yang L, Marmolejo DC, Martí CP (2018). Identifying the urban space for locals and tourists through 'Foursquare' data in Barcelona. International conference virtual city and territory. 10.5821/ctv.8238

[CR69] Yang L, Marmolejo DC (2019). Identifying tourist-functional relations of urban places through Foursquare from Barcelona. GeoJournal.

[CR70] Yun HJ, Kang DJ, Lee MJ (2018). Spatiotemporal distribution of urban walking tourists by season using GPS-based smartphone application. Asia Pac J Tour Res.

[CR71] Zhang J, Teng C-Y, Qu Y (2013) Understanding user spatial behaviors for location-based recommendations. En WWW'13: 22nd international conference on World Wide Web. pp 989–992. 10.1145/2487788.2488096

[CR72] Zhong C, Schläpfer M, Müller A, Stefan B, Michael RC, Schmitt G (2017). Revealing centrality in the spatial structure of cities from human activity patterns. Urban Stud.

