# Supplementary material for: Scoping out urban areas of tourist interest though geolocated social media data: Bucharest as a case study
Source: Inf Technol Tourism. 2022 Oct 17;24(3):361–87. doi: 10.1007/s40558-022-00235-8 (PMC9574161; doi:10.1007/s40558-022-00235-8)
Supplement: Supplementary file 1 — Supplementary file1 (PDF 9922 kb) [file 40558_2022_235_MOESM1_ESM.pdf]

Supplementary material: **Corresponding TripAdvisor Categories and Subcategories to Foursquare Categories and Subcategories.**

TripAdvisor Categories	TripAdvisor Subcategories [found in Bucharest Dataset]	Foursquare Subcategory	Foursquare Category
Casinos & Gaming	Casinos	Casino	Arts & Entertainment
Classes & Workshops	Classes & Workshops	General Entertainment	
	Cooking Classes		
	Lessons & Workshops		
Fun & Games	Room Escape Games		
	Game & Entertainment Centres	Bowling Alley	
		Go Kart Track	
		Circus	
		Laser Tag	
	Movie Theatres	Movie Theater	
	Sports Complexes	Pool Hall	
	Mini Golf	Mini Golf	
	Comedy Clubs	Comedy Club	
Concerts & Shows	Theatres	Amphitheater	
	Concerts	Concert Hall	
	Blues Clubs & Bars	Music Venue	
	Jazz Clubs & Bars		
	Piano Bars		
	Symphonies		
	Performances	Performing Arts Venue	
Museums	Art Museums	Museum	
	Children's Museums		
	History Museums		
	Natural History Museums		
	Science Museums		
	Speciality Museums		
	Art Galleries	Art Gallery	
Shopping	Fashion Shows & Tours	Tour Provider	
	Shopping Tours		
Boat Tours & Water Sports	Boat Tours		
Outdoor Activities	4WD, ATV & Off-Road Tours		
	Adrenaline & Extreme Tours		
	Air Tours		
	Bike Tours		
	Canyoning & Rappelling Tours		
	Climbing Tours		
	Hiking & Camping Tours		
	Horseback Riding Tours		
	Horse-Drawn Carriage Tours		
	Nature & Wildlife Tours		
	Running Tours		
	Ski & Snow Tours		
		Racecourse	
		Racetrack	
Sights & Landmarks		Public Art	
	Historic Sites	Historic Site	
	Monuments & Statues	Memorial Site	
	Arenas & Stadiums	Stadium	
Water & Amusement Parks	Theme Parks	Theme Park	
	Water Parks	Water Park	
Zoos & Aquariums	Zoos	Aquarium	
Events		Festival	Event
		Other Event	
		Parade	
Shopping	Flea & Street Markets	Street Fair	
Traveler Resources	Conference & Convention Centres	Conference	
Boat Tours & Water Sports	Scuba & Snorkeling	Dive Spot	Outdoors & Recreation
Nature & Parks		Beach	
		Mountain	
		Other Great Outdoors	
	Gardens	Botanical Garden	
		Garden	
	Bodies of Water	Lake	
		River	
	Nature & Wildlife Areas	Nature Preserve	
	Parks	Park	
	Biking Trails	Trail	
	Equestrian Trails		
	Hiking Trails		
	Playgrounds	Playground	
Fun & Games		Indoor Play Area	
	Shooting Ranges	Gun Range	
	Sports Complexes	Pool	
		Recreation Center	
	Game & Entertainment Centres	Rock Climbing Spot	
Outdoor Activities	Climbing Tours		
	Hiking Trails	Trail	
	Equestrian Trails		
		Dog Run	
		Ski Area	
Classes & Workshops	Sports Camps & Clinics	Athletics & Sports	
Spas & Wellness	Health/Fitness Clubs & Gyms		
Sights & Landmarks	Points of Interest & Landmarks	Bathing Area	
		Canal	
		Harbor / Marina	
		Pedestrian Plaza	
		Plaza	
	Bridges	Bridge	
	Castles	Castle	
	Cemeteries	Cemetery	
	Fountains	Fountain	
		Island	
		Lighthouse	
		Palace	
		Volcano	
		Waterfront	
	Observation Decks & Towers	Roof Deck	
	Lookouts	Scenic Lookout	
	Monuments & Statues	Sculpture Garden	
Classes & Workshops	Paint & Pottery Studios	Art Studio	Professional & Other Places
Concerts & Shows	Operas	Auditorium	
Fun & Games		Fair	
Museums	Observatories & Planetariums	Observatory	
Sights & Landmarks	Observation Decks & Towers		
	Architectural Buildings	Building	
	Civic Centres	Community Center	
		Cultural Center	
	Government Buildings	Government Building	
	Churches & Cathedrals	Spiritual Center	
	Sacred & Religious Sites		
Traveler Resources	Conference & Convention Centres	Convention Center	
	Libraries	Library	
Boat Tours & Water Sports	Scuba & Snorkeling	Dive Shop	Shop & Service
Classes & Workshops	Paint & Pottery Studios	Arts & Crafts Store	
Fun & Games	Casinos	Betting Shop	
Nature & Parks	Gardens	Garden Center	
Outdoor Activities	Bike Tours	Bike Shop	
		Auto Dealership	
		Board Shop	
Traveler Resources		Currency Exchange	
		Travel Agency	
Shopping		Market	
	Antique Stores	Antique Shop	
	Farmers Markets	Flea Market	
	Flea & Street Markets		
	Factory Outlets	Outlet Mall	
	Shopping Malls	Shopping Mall	
	Gift & Speciality Shops	Gift Shop	
		Souvenir Shop	
Spas & Wellness	Hammams & Turkish Baths	Bath House	
		Health & Beauty Service	
	Yoga & Pilates		
	Health/Fitness Clubs & Gyms	Massage Studio	
	Spas	Spa	
Boat Tours & Water Sports	Boat Hire	Cruise	Travel & Transport
Outdoor Activities	Boat Tours		
	Bike Tours	Bike Rental / Bike Share	
		Rental Car Location	
Sights & Landmarks		Pier	
Traveler Resources		General Travel	
		Rest Area	
		Travel Lounge	
	Visitor Centres	Tourist Information Center	
Transportation	Bus Transportation	Bus Station	
		Bus Stop	
	Mass Transportation Systems	Light Rail Station	
		Metro Station	
		Train Station	
	Taxis & Shuttles	Taxi	
		Taxi Stand	
		Transportation Service	
